# Chromosome number evolution in skippers (Lepidoptera, Hesperiidae)

**DOI:** 10.3897/CompCytogen.v8i4.8789

**Published:** 2014-11-14

**Authors:** Vladimir A. Lukhtanov

**Affiliations:** 1 Department of Entomology, Faculty of Biology, St. Petersburg State University, Universitetskaya nab. 7/9, 199034 St. Petersburg, Russia; 2 Department of Karyosystematics, Zoological Institute of Russian Academy of Science, Universitetskaya nab. 1, 199034 St. Petersburg, Russia

**Keywords:** Lepidoptera, Hesperiidae, karyotype evolution, chromosome number, cryptic species, phylogeny, chromosomal conservatism, chromosomal instability

## Abstract

Lepidoptera (butterflies and moths), as many other groups of animals and plants, simultaneously represent preservation of ancestral karyotype in the majority of families with a high degree of chromosome number instability in numerous independently evolved phylogenetic lineages. However, the pattern and trends of karyotype evolution in some Lepidoptera families are poorly studied. Here I provide a survey of chromosome numbers in skippers (family Hesperiidae) based on intensive search and analysis of published data. I demonstrate that the majority of skippers preserve the haploid chromosome number n=31 that seems to be an ancestral number for the Hesperiidae and the order Lepidoptera at whole. However, in the tribe Baorini the derived number n=16 is the most typical state which can be used as a (syn)apomorphic character in further phylogenetic investigations. Several groups of skippers display extreme chromosome number variations on within-species (e.g. the representatives of the genus *Carcharodus* Hübner, [1819]) and between-species (e.g. the genus *Agathymus* Freeman, 1959) levels. Thus, these groups can be used as model systems for future analysis of the phenomenon of chromosome instability. Interspecific chromosomal differences are also shown to be useful for discovering and describing new cryptic species of Hesperiidae representing in such a way a powerful tool in biodiversity research. Generally, the skipper butterflies promise to be an exciting group that will significantly contribute to the growing knowledge of patterns and processes of chromosome evolution.

## Introduction

The main karyotypic features of organisms, particularly the number of chromosomes, tend to be stable within species (White 1973, King 1993). New chromosomal rearrangements usually originate as heterozygotes and are often – although not always (Lukhtanov et al. 2011) – associated with heterozygote disadvantage. The spread of such rearrangements to fixation within a large population has low probability (King 1993). Therefore, many organisms are characterized by chromosomal conservatism, a situation in which all closely related taxa demonstrate the same chromosome number.

In contrast to chromosomal conservatism, chromosomal instability characterizes situations where multiple closely related taxa (populations, subspecies and/or species) belonging to a single phylogenetic lineage differ drastically from each other by major chromosomal rearrangements, sometimes resulting in high variability in chromosome number.

Both phenomena - chromosomal conservatism and chromosomal instability - are clearly expressed in insects of the order Lepidoptera (butterflies and moths). The modal haploid number of chromosomes (n) of n = 31 or n = 30 (Suomalainen 1969, Lukhtanov 2000) is preserved in the majority of lepidopteran families (Robinson 1971). At the same time, numerous cases of chromosomal instability have been discovered in the butterfly families, e.g. in Papilionidae (Emmel et al. 1995), Pieridae (Lukhtanov 1991, Lukhtanov et al. 2011, Dinca et al. 2011), Nymphalidae (Brown et al. 1992, 2004, 2007a, 2007b) and Riodinidae (Brown et al. 2012). This phenomenon was analyzed in more detail in the family Lycaenidae (Kandul et al. 2004, 2007, Lukhtanov et al. 2005, 2006, 2008, Vershinina and Lukhtanov 2010, 2013, Vila et al. 2010, Talavera et al. 2013, Przybyłowicz et al. 2014).

Skippers (the family Hesperiidae) are studied to a lesser extent with the respect of karyotype evolution than the other butterfly families mentioned above (but see: Emmel and Trew 1973, Saura et al. 2013). This family includes about 4000 species under 567 genera and is a globally distributed group found in all continents except Antarctica (Warren et al. 2008). The tribal level classification of skippers, based on combined analysis of molecular and morphological data, was recently elaborated by Warren and colleagues (Warren et al. 2008, 2009).

Here I provide a first world-wide survey of chromosome numbers in skippers based on intensive search and analysis of published data.

## Results

The results of literature search are presented in the Table below. It includes all the discovered chromosome counts except n=13 for *Ochlodes venatus* (Bremer et Grey, 1853), noted by Bigger (1960) as “*Augiades venata*”. The name *Ochlodes venatus* was long used for the *Ochlodes* species of Europe, but it actually refers to its Far Eastern sister species, and the European taxon is now called *Ochlodes sylvanus* (Esper, 1777) (ICZN 2000). Both European and Far Eastern species have the same chromosome number n=29 (Federley 1938, Lorković 1941, Abe et al. 2006), not n=13 as indicated by Bigger (1960). Thus, the species name used by Bigger (1960) was probably misidentification.

The classification of skippers accepted in this paper follows Warren and colleagues (Warren et al. 2008, 2009).

## Discussion

### Modal chromosomal numbers

The table gives the chromosome numbers of 205 species of skippers, i.e. about 5% of the species of the world fauna. This number is not enough to infer any final statements about peculiarities of chromosome numbers distribution within the Hesperiidae. However, several tentative conclusions can be made. The haploid chromosome number n=31 was found in 50 studied species of skippers and, thus, it is a clear modal number for the family at whole. Interestingly, n=31 was found in representatives of all investigated subfamilies, except for Heteropterinae. However, in the last subfamily only one species was karyologically studied until now, and discovery of n=31 in Heteropterinae is not excluded in future. The next most common numbers are n=29 (43 species), n=30 (33 species) and n=28 (13 species).

Subfamilies Coeliadinae and Eudaminae have a sharp peak at n=31. In the subfamily Trapezitinae n=31 was also found (only one species studied).

Within the subfamily Pyrginae, the modal number n=31 is found in the tribe Erynnini. The tribe Pyrrhopygini is characterized by the most common n=28. The modal number in the tribe Tagiadini is n=30. The tribe Carcharodini has peaks at n=30 and n=31. In the tribe Pyrgini, n=29, n=30 and n=31 were found as the most common numbers.

In the family Heteropterinae n=29 was found (only one species studied).

Within the subfamily Hesperiinae, the tribes Taractrocerini, Thymelicini, Calpodini, Moncini and Hesperiini are characterized by the most common n=29. Very variable chromosome numbers (from n=5 to n=50) were found in the tribe Aeromachini. It is difficult to infer the modal number for the last tribe. However, it should be noted that one species, *Thoressa varia*, has n=31 as the majority of other skippers. The tribe Baorini (subfamily Hesperiinae) has a clear peak at n=16, so it is exceptional with respect to the modal number of chromosomes.

The overall evidence indicates that chromosome numbers of Coeliadinae, Eudaminae, Trapezitinae, Pyrginae and Hesperiinae conform to the lepidopteran modal of n=31 (Robinson 1971). This number seems to be an ancestral one for the Hesperiidae as for the order Lepidoptera at whole (Suomalainen 1969, Lukhtanov 2000). This modal number (or its deviation to n=30, n=29 and 28) were preserved in the majority of skippers. However, in the tribe Baorini the number n=16 was evolved and, thus, represents a derived trait which can be used as a (syn)apomorphic character in further phylogenetic studies of the family Hesperiidae.

### Between- and within-species variations in chromosome number

Several groups of skippers display extreme chromosome number variations at the within-species level (Table). The most extreme variations in number of chromosome elements were observed in first meiotic metaphase of *Carcharodus boeticus*, *Carcharodus dravira* and *Carcharodus flocciferus* (Table, de Lesse 1960). The nature of these variations remains unknown, and there are two plausible explanations for this phenomenon. First, this variation can be explained by presence of so-called B-chromosomes (=additional chromosomes, =supernumerary chromosomes) (de Lesse 1960). B-chromosomes consist mainly of repetitive DNA and can sometimes accumulate through processes of mitotic or meiotic drive (Jones et al. 2008). B-chromosomes can be distinguished from normal A-chromosomes because they are usually smaller and can be seen as additional chromosomes present in only some of the individuals in a population (Camacho et al. 2000, Jones et al. 2008).

Second, this kind of variation can be caused by violations in meiotic chromosome pairing resulting in appearance of univalents (instead of bivalents) in meiotic prophase (Lorković 1990). This type of variation was studied in detail by Maeki and Ae (1979) in butterfly genus *Papilio* and is expected if regular or irregular interspecific mating occurs in nature. Anyway, the nature of intraspecific variations observed in *Carcharodus* is different from that discovered in the Wood White butterfly *Leptidea sinapis* (Linnaeus, 1758) (Pieridae). In the last species the compared range of intraspecific variation in chromosome number (from n=28 to n=53) was caused by multiple chromosome fusions/fissions accumulated within the species (Lukhtanov et al. 2011, Dinca et al. 2011).

Between-species variation exists in numerous genera of skippers (Table 1) and is especially expressed in the Nearctic genus *Agathymus* Freeman, 1959, in which the range of haploid numbers was discovered from n =5 in *Agathymus aryxna* to n=38 in *Agathymus alliae* (Freeman 1969). This range is comparable of even exceeds the range found in chromosomally diverse genera from other butterfly families (Lorković 1990, Lukhtanov et al. 2005, Talavera et al. 2013). Thus, the genera of Hesperiidae can be used as model systems for future analysis of the phenomenon of chromosome instability.

**Table 1.** Chromosome number of skippers (Lepidoptera, Hesperiidae) of the world fauna (Us are univalents; 2n is diploid chromosome number). <br/> Years of the species descriptions are given square brackets in cases where they were not stated in the original sources but were inferred from reliable external evidence.

#	Species	Haploid chromosome number	Country	Reference
**Subfamily Coeliadinae**				
1	*Bibasis aquilina* (Speyer, 1879)	29	Japan	Maeki 1953
	*Bibasis aquilina chrysaeglia* (Butler, 1881)	31 (2n=62)	Japan	Abe et al. 2006
2	*Bibasis jaina formosana* Fruhstorfer, 1911	31	Taiwan	Maeki and Ae 1968b
3	*Choaspes benjaminii* (Guérin-Méneville, 1843)	31	Japan	Maeki 1953
	*Choaspes benjaminii japonica* (Murray, 1875)	31	Japan	Saitoh et al. 1978
4	*Coeliades anchises jucunda* (Butler, 1881)	30	Oman	Saitoh 1982
5	*Coeliades ernesti* (Grandidier, 1867)	31	Madagascar	de Lesse 1972
6	*Coeliades fervida* (Butler, 1880)	23	Madagascar	de Lesse 1972
7	*Coeliades forestan arbogastes* (Guenee, 1863)	31	Madagascar	de Lesse 1972
8	*Coeliades ramanatek* (Boisduval, 1833)	31	Madagascar	de Lesse 1972
**Subfamily Euschemoninae** no chromosomal data available				
**Subfamily Eudaminae**				
9	*Achalarus casica* (Herrich-Schäffer, 1869)	29	USA (Texas)	Emmel and Trew 1973
10	*Achalarus lyciades* (Geyer, 1832)	31	USA (Connecticut)	Maeki 1961
11	*Achalarus toxeus* (Plötz, 1882)	16	Mexico	Maeki and Remington 1960
12	*Astraptes anaphus* (Godman et Salvin, 1896)	31	Bolivia	de Lesse 1967a
13	*Astraptes fulgerator* (Walch, 1775)	31	Peru	Kumagai et al. 2010
14	*Astraptes naxos* (Hewitson, 1867)	31	Brazil	Saura et al. 2013
15	*Astraptes phalaecus* (Godman et Salvin, 1893)	25	Guatemala	de Lesse 1967a
16	*Astraptes longipennis* (Plötz, 1882)	31	Costa Rica	Kumagai et al. 2010
		31	Peru	Kumagai et al. 2010
		31	Brazil	Kumagai et al. 2010
17	*Autochton* sp.	20, 21	Brazil	Kumagai et al. 2010
18	*Chioides albofasciatus* (Hewitson, 1867)	31	Mexico	de Lesse 1970a
	*Chioides albofasciatus* (Hewitson, 1867) (as *Chioides catillus*)	31	Mexico	Maeki and Remington 1960
	*Chioides albofasciatus* (Hewitson, 1867)	31	USA (Texas)	Emmel and Trew 1973
19	*Entheus priassus pralina* Evans, 1952	22	Brazil	Saura et al. 2013
20	*Epargyreus barisses* (Hewitson, 1874)	31	Argentina	de Lesse 1967
21	*Epargyreus clarus* (Cramer, 1775)	31	USA (Florida)	Maeki 1961
22	*Epargyreus clavicornis tenda* Evans, 1955	ca 29–30	Guatemala	de Lesse 1970a
23	*Oechydrus chersis* (Herrich-Schäffer, 1869)	31	Bolivia	de Lesse 1967a
24	*Phocides polybius phanias* (Burmeister, 1880)	16	Brazil	Saura et al. 2013
25	*Tarsoctenus praecia plutia* (Hewitson, 1857)	15	Brazil	Saura et al. 2013
26	*Thorybes pylades pylades* (Scudder, 1870)	31	USA (Connecticut)	Maeki 1961
27	*Udranomia spitzi* (Hayward, 1942)	29	Brazil	de Lesse and Brown 1971
28	*Urbanus dorantes dorantes* (Stoll, 1790)	31	Mexico	de Lesse 1970a
29	*Urbanus doryssus doryssus* (Swainson, 1831)	14	Costa Rica	Kumagai et al. 2010
30	*Urbanus proteus* (Linnaeus, 1758)	31	Bolivia	de Lesse 1967a
		31	Mexico	de Lesse 1970a
		31	USA (Florida)	Maeki 1961
31	*Urbanus simplicius* (Stoll, 1790)	31	Argentina	de Lesse 1967a
32	*Urbanus teleus* (Hübner, 1821)	31	Argentina	de Lesse 1967a
**Subfamily Pyrginae**				
**Tribe Pyrrhopygini**				
33	*Elbella lamprus* (Hopffer, 1874)	40	Brazil	de Lesse 1970a
34	(?) *Jemadia* sp.	32(?)	Brazil	Saura et al. 2013
35	*Mimoniades montana* J. Zikán, 1938	27	Brazil	Saura et al. 2013
36	*Mimoniades nurscia* (Swainson, 1821)	28	Ecuador	de Lesse 1967a
	*Mimoniades nurscia malis* (Godman et Salvin, 1879)	28	Colombia	Saura et al. 2013
37	*Mimoniades* sp.	21	Colombia	Saura et al. 2013
38	*Mimoniades* sp.	28	Colombia	Saura et al. 2013
39	*Mimoniades versicolor* (Latreille, [1824])	28	Brazil	de Lesse and Brown 1971
40	*Pyrrhopyge charybdis* Westwood, 1852	14(?)	Brazil	Saura et al. 2013
41	*Pyrrhopyge pelota* Plötz, 1879	28	Argentina	de Lesse 1967a
42	*Pyrrhopyge* sp.	15	Brazil	Saura et al. 2013
43	*Sarbia* sp.	30	Brazil	Saura et al. 2013
**Tribe Tagiadini**				
44	*Daimio tethys* (Ménétriés, 1857)	30	Japan	Maeki 1953, Maeki and Makino 1953
45	*Daimio tethys moorei* Mabille, 1876	30	Taiwan	Maeki and Ae 1968b
46	*Eagris lucetia* (Hewitson, 1876)	30	Uganda	de Lesse 1968
47	*Eagris sabadius astoria* Holland, 1896	30	Kenya	de Lesse 1968
48	*Eretis lugens* (Rogenhofer, 1891	28	Kenya	de Lesse 1968
**Tribe Celaenorrhinini**				
49	*Sarangesa phidyle* (Walker, 1870)	29	Senegal	de Lesse and Condamin 1962
**Tribe Carcharodini**				
50	*Carcharodus alceae* (Esper, [1780])	31	Croatia	Lorković 1941
51	*Carcharodus boeticus* Reverdin, 1913	43–47	Spain	de Lesse 1960
	*Carcharodus boeticus* Reverdin, 1913	40–52	France	de Lesse 1960
	*Carcharodus boeticus* Reverdin, 1913	38–46	Italy	de Lesse 1960
52	*Carcharodus dravira* (Moore, 1874)	37–48 (with Us)	Iran	de Lesse 1960
53	*Carcharodus flocciferus* (Zeller, 1847)	32–41 (with Us)	France (Cauterets)	de Lesse 1960
54	*Carcharodus flocciferus* (Zeller, 1847)	42–58 (with Us)	Italy	de Lesse 1960
55	*Carcharodus lavatherae* (Esper, [1783])	30	France (Salau, Ariege)	de Lesse 1960
56	*Carcharodus orientalis* Reverdin, 1913	31–32	Lebanon	de Lesse 1960
		30	Turkey (Van)	de Lesse 1960
		30–37 (with Us)	Turkey (Amasya)	de Lesse 1960
57	*Carcharodus stauderi ambiguus* Verity, 1925	30	Lebanon	de Lesse 1960
		30	Turkey	de Lesse 1960
58	*Hesperopsis alpheus* (W. H. Edwards, 1876) (as *Pholisora*)	34	USA (Texas)	Emmel and Trew 1973
59	*Muschampia nomas* (Lederer, 1855)	30	Lebanon	de Lesse 1960
60	*Muschampia proteides* (Wagner, 1929)	30	Lebanon	Larsen 1975
61	*Muschampia proto* (Ochsenheimer, 1808)	30	Spain	de Lesse 1960
		30	Lebanon	Larsen 1975
62	*Pholisora catullus* (Fabricius, 1793)	29	?USA	Lorković in Robinson 1971
63	*Spialia orbifer* (Hübner, [1823])	30	Croatia	Lorković 1941
		31	Turkey	de Lesse 1960
64	*Spialia phlomidis* (Herrich-Schäffer, [1845])	31	Turkey	de Lesse 1960
65	*Spialia sertorius* (Hoffmannsegg, 1804)	31	Slovenia	Lorković 1941
**Tribe Erynnini**				
66	*Chiomara asychis georgina* (Reakirt, 1868)	31	Mexico	de Lesse 1970a
	*Chiomara asychis georgina* (Reakirt, 1868)	32	USA (Texas)	Emmel and Trew 1973
67	*Chiomara* sp.	31	Trinidad	Wesley and Emmel 1975
68	*Ebrietas anacreon* (Staudinger, 1876)	31	Argentina	de Lesse 1967a
69	*Ebrietas osyris* (Staudinger, 1876)	31	Argentina	de Lesse 1967a
70	*Erynnis baptisiae* (W. Forbes, 1936)	31	USA (Connecticut)	Maeki 1961
71	*Erynnis funeralis* (Scudder et Burgess, 1870)	31	Argentina	de Lesse 1967a
72	*Erynnis horatius* (Scudder et Burgess, 1870)	31	USA (Florida)	Maeki 1961
73	*Erynnis icelus* (Scudder et Burgess, 1870)	30	USA (Connecticut)	Maeki 1961
74	*Erynnis juvenalis juvenalis* (Fabricius, 1793)	30	USA (Connecticut)	Maeki 1961
75	*Erynnis lucilius* (Scudder et Burgess, 1870)	31	USA (Connecticut)	Maeki and Remington 1960a
76	*Erynnis marloyi* (Boisduval, [1834])	31	Lebanon	de Lesse 1960
77	*Erynnis montanus* (Bremer, 1861)	31 (2n=62)	Japan	Abe et al. 2006
	*Erynnis montanus* (Bremer, 1861)	31	Japan	Maeki 1953
78	E. *persius* (Scudder, 1863)	31	USA (Connecticut)	Maeki 1961
79	*Erynnis tages* (Linnaeus, 1758)	31	Croatia	Lorković 1941
		31	France	de Lesse 1960
		31	England	Bigger 1960
80	*Erynnis tristis tatius* (W. H. Edwards, 1883)	31	USA (Texas)	Emmel and Trew 1973
81	*Gesta gesta* (Herrich-Schäffer, 1863)	32	Tobago	Wesley and Emmel 1975
82	*Grais stigmaticus* (Mabille, 1883)	31	Mexico	Maeki and Remington 1960a
83	*Theagenes albiplaga* (C. Felder et R. Felder, 1867)	31	Bolivia	de Lesse 1967a
**Tribe Achlyodidini**				
84	*Achlyodes pallida* (R. Felder, 1869) (as *Achlyodes selva*)	15	Bolivia	de Lesse 1967a
		15	Mexico	de Lesse 1970a
85	*Zera zera zera* (Butler, 1870)	34	Brazil	de Lesse and Brown 1971
**Tribe Pyrgini**				
86	*Anisochoria sublimbata* Mabille, 1883	31	Argentina	de Lesse 1967a
87	*Antigonus erosus* (Hübner, [1812])	31	Mexico	de Lesse 1970a
88	*Antigonus liborius* Plötz, 1884	31	Argentina	de Lesse 1967a
89	*Celotes nessus* (W. H. Edwards, 1877)	14, 13	USA (Texas)	Emmel and Trew 1973
90	*Heliopetes arsalte* (Linnaeus, 1758)	30	Bolivia	de Lesse 1967a
	*Heliopetes arsalte* (Linnaeus, 1758)	30	Mexico	de Lesse 1970a
91	*Heliopetes laviana* (Hewitson, 1868)	29	USA (Texas)	Emmel and Trew 1973
92	*Heliopetes macaira* (Reakirt, [1867])	29	USA (Texas)	Emmel and Trew 1973
93	*Heliopetes omrina* (Butler, 1870)	30	Argentina	de Lesse 1967a
94	*Heliopyrgus americanus* (Blanchard, 1852)	30	Chile	de Lesse 1967a
95	*Paches loxus* (Westwood, [1852])	31	Guatemala	de Lesse 1970a
96	*Pyrgus aladaghensis* De Prins et van der Poorten, 1995	ca 18–21	Turkey	Lukhtanov and Kandul 1995 (in Hesselbarth et al. 1995)
97	*Pyrgus albescens* Plötz, 1884	30 (2n=60)	USA (Texas)	Goodpasture 1976
	*Pyrgus albescens* Plötz, 1884	28	USA (Texas)	Emmel and Trew 1973
98	*Pyrgus alveus* (Hübner, [1803])	24	Finland	Federley 1938
		24	Croatia	Lorković 1941
		24	Turkey	Lukhtanov and Kandul 1995 (in Hesselbarth et al. 1995)
99	*Pyrgus bellieri* (Oberthür, 1910)	27	France	de Lesse 1960
100	*Pyrgus bocchoris* (Hewitson, 1874)	30	Argentina	de Lesse 1967a
101	*Pyrgus bolkariensis* De Prins et van der Poorten, 1995	30	Turkey	Lukhtanov and Kandul 1995 (in Hesselbarth et al. 1995)
102	*Pyrgus cacaliae* (Rambur, 1839)	30	Italy	de Lesse 1960
103	*Pyrgus carlinae* (Rambur, [1839])	30	Italy	de Lesse 1960
104	*Pyrgus carthami* (Hübner, [1813])	29	Italy	de Lesse 1960
105	*Pyrgus cirsii* (Rambur, [1839])	30	France (Peyreleau, Aveyron)	de Lesse 1960
106	*Pyrgus fides* Hayward, 1940	30	Chile	de Lesse 1967a
107	*Pyrgus maculates* (Bremer et Grey, 1852)	31 (2n=62)	Japan	Abe et al. 2006
108	*Pyrgus malvae* (Linnaeus, 1758)	31	Finland	Federley 1938
		33	England	Bigger 1960
109	*Pyrgus oileus* (Linnaeus, 1767)	30 (2n=60)	USA (Texas)	Goodpasture 1976
		32	USA (Texas)	Emmel and Trew 1973
110	*Pyrgus onopordi* (Rambur, [1839])	30	France	Lorković 1941
111	*Pyrgus serratulae* (Rambur, [1839])	30	France	Lorković 1941
112	*Trina geometrina geometrina* (C. Felder et R. Felder, 1867)	31	Brazil	de Lesse and Brown 1971
**Subfamily Heteropterinae**				
113	*Butleria quilla* Evans, 1939	29	Chile	de Lesse 1967a

**Subfamily Trapezitinae**				
114	*Trapezites eliena* Hewitson, 1868	31	Australia	Maeki and Ogata 1971
**Subfamily Hesperiinae**				
**Tribe Aeromachini**				
115	*Aegiale hesperiaris* (Walker, 1856)	24	Mexico	Freeman 1969
116	*Agathymus alliae* (Stallings et Turner, 1957)	38	USA (Arizona)	Freeman 1969
117	*Agathymus aryxna* (Dyar, 1905)	5	Mexico	Freeman 1969
118	*Agathymus baueri* (Stallings et Turner, 1954)	15	USA (Arizona)	Freeman 1969
119	*Agathymus chisosensis* (Freeman, 1952)	18	USA (Texas)	Freeman 1969
120	*Agathymus estelleae valverdiensis* Freeman, 1966	9	USA (Texas)	Freeman 1969
	*Agathymus estelleae estelleae* (Stallings et Turner, 1958)	9	Mexico	Freeman 1969
121	*Agathymus freemani* Stallings, Turner et Stallings, 1960	15	USA (Arizona)	Freeman 1969
122	*Agathymus gilberti* Freeman, 1964	21	USA (Texas)	Freeman 1969
123	*Agathymus mariae chinatiensis* Freeman, 1964	22	USA (Texas)	Freeman 1969
	*Agathymus mariae lajitaensis* Freeman, 1964	22	USA (Texas)	Freeman 1969
	*Agathymus mariae mariae* (Barnes et Benjamin, 1924)	22	USA or Mexico	Freeman 1969
	*Agathymus mariae rindgei* Freeman, 1964	22	USA (Texas)	Freeman 1969
124	*Agathymus micheneri* Stallings, Turner et Stallings, 1961	20	Mexico	Freeman 1969
125	*Agathymus neumoegeni florenceae* (Stallings et Turner, 1957)	10	USA (Texas)	Freeman 1969
	*Agathymus neumoegeni macalpinei* (Freeman, 1955)	10	USA (Texas)	Freeman 1969
126	*Agathymus polingi* (Skinner, 1905)	10	USA (Arizona)	Freeman 1969
127	*Agathymus remingtoni* (Stallings et Turner, 1958)	9	Mexico	Freeman 1969
128	*Alera vulpina* (C. Felder et R. Felder, 1867)	ca27	Ecuador	de Lesse 1967a
129	*Ankola fan* (Holland, 1844)	10	Uganda	De Lesse 1968
130	*Arotis derasa* (Herrich-Schäffer, 1870) (as *Euphyes*)	28	Brazil	de Lesse and Brown 1971
131	*Erionota thrax thrax* (Linnaeus, 1767)	29	Malaysia	Saitoh and Kumagai 1974
132	*Euphyes leptosema* Mabille, 1891	ca28	Argentina	de Lesse 1967a
133	*Megathymus coloradensis coloradensis* Riley, 1877	27	USA	Freeman 1969
134	*Megathymus coloradensis kendalli* Freeman, 1965	27	USA (South central Texas)	Freeman 1969
	*Megathymus coloradensis louiseae* Freeman, 1963	27	USA (Western Texas)	Freeman 1969
	*Megathymus coloradensis navajo* Skinner, 1911	27	USA	Freeman 1969
	*Megathymus coloradensis reinthali* Freeman, 1963	27	USA (Texas)	Freeman 1969
	*Megathymus coloradensis reubeni* Stallings, Turner et Stallings, 1963	27	USA (Texas)	Freeman 1969
	*Megathymus coloradensis stallingsi* Freeman, 1943	27	USA	Freeman 1969
	*Megathymus coloradensis wilsonorum* Stallings et Turner, 1958	27	?Mexico	Freeman 1969
135	*Megathymus violae* Stallings et Turner, 1956	27	USA	Maeki 1961, Freeman 1969
136	*Megathymus yuccae buchholzi* Freeman, 1952	26	USA (Florida)	Freeman 1969
137	*Pardaleodes incerta* (Snellen, 1872)	17	Uganda	de Lesse 1968
138	*Stallingsia maculosus* (Freeman, 1955)	50	USA (Texas)	Maeki 1961, Freeman 1969
139	*Suastus gremius* (Fabricius, 1798)	23	Taiwan	Maeki and Ae 1968b
140	*Thoressa varia* (Murray, 1875)	31 (2n=62)	Japan	Abe et al. 2006
141	*Thoressa varia* (Murray, 1875)	31	Japan	Maeki 1953
**Tribe Baorini**				
142	*Gegenes gambica* (Mabille, 1878)	41	Yemen	Saitoh 1984
		41	Turkey	de Lesse 1960
		41	Lebanon	Larsen 1982
143	*Gegenes nostrodamus* (Fabricius, 1793)	15	Egypt	Larsen 1982
		15	Israel	Saitoh 1979, Larsen 1982
144	*Gegenes pumilio* (Hoffmansegg, 1804)	24	France	de Lesse 1960
		24	Alger	de Lesse 1967b
145	*Parnara guttata* (Bremer et Grey, 1852)	16	Japan	Maeki 1953, Maeki and Makino 1953
		16	China	Saitoh and Abe 1981
146	*Pelopidas conjucta conjucta* (Herrich-Schäffer, 1869)	16	Hong Kong	Maeki and Ae 1968a
147	*Pelopidas jansonis* (Butler, 1878)	16 (2n=32)	Japan	Abe et al. 2006
148	*Pelopidas mathias* (Fabricius, 1798)	16	Japan	Maeki and Remington 1960
149	*Pelopidas thrax* (Hübner, [1821])	16	Lebanon	Larsen 1975
150	*Polytremis lubricans* (Herrich-Schäffer, 1869)	16	Taiwan	Maeki and Ae 1968b
151	*Polytremis pellucida* (Murray, 1875)	16, 17, 18 (2n=32, 33)	Japan	Abe et al. 2006
		16	Japan	Maeki and Remington 1960
152	*Zenonia zeno* (Trimen, 1864)	16	Uganda	de Lesse 1968
**Tribe Taractrocerini**				
153	*Ocybadistes walkeri sothis* Waterhouse, 1933	28	Australia	Maeki and Ogata 1971
154	*Potanthus flavus* (Murray, 1875)	29 (2n=58)	Japan	Abe et al. 2006
155	*Telicota ancilla horisha* Evans, 1934	29	Taiwan	Maeki and Ae 1968b
156	*Telicota colon stinga* Evans, 1949	29	Japan (Okinava)	Abe et al. 2006
157	*Telicota ohara formosana* Fruhstorfer, 1911	29 (2n=58)	Taiwan	Abe et al. 2006
**Tribe Thymelicini**				
158	*Copaeodes minima* (W.H. Edwards, 1870)	29	USA (Florida)	Maeki 1961
159	*Thymelicus sylvestris* (Poda, 1761)	27	England	Bigger 1960
160	*Thymelicus sylvaticus* (Bremer, 1861)	10 (2n=20)	Japan	Abe et al. 2006
161	*Thymelicus acteon* (Rottemburg, 1775)	28	Spain	de Lesse 1970c
162	*Thymelicus hyrax* (Lederer, 1861)	29	Lebanon	Larsen 1975
163	*Thymelicus leoninus* (Butler, 1878)	9 (2n=18)	Japan	Abe et al. 2006
164	*Thymelicus lineola* (Ochsenheimer, 1808)	29	Finland	Federley 1938
		29	Lebanon	Larsen 1975
**Tribe Calpodini**				
165	*Ebusus ebusus* (Cramer, [1780])	29	Mexico	de Lesse 1970a
166	*Lychnuchus celsus* (Fabricius, 1793)	30	Brazil	de Lesse and Brown 1971
167	*Panoquina hecebolus* (Scudder, 1872)	29	USA (Texas)	Emmel and Trew 1973
168	*Panoquina ocola* (W. H. Edwards, 1863)	29	USA (Texas)	Emmel and Trew 1973
169	*Panoquina panoquin* (Scudder, 1863)	29	USA (Florida)	Maeki 1961
170	*Panoquina panoquinoides* (Skinner, 1891)	29	USA (Texas)	Emmel and Trew 1973
**Tribe Anthoptini** no chromosomal data available				
**Tribe Moncini**				
171	*Amblyscirtes aenus* W.H. Edwards, 1878	28, 29	USA (Texas)	Emmel and Trew 1973
172	*Amblyscirtes cassus* W. H. Edwards, 1883	29	USA (Texas)	Emmel and Trew 1973
173	*Amblyscirtes celia* (Skinner, 1895)	29	USA (Texas)	Emmel and Trew 1973
174	*Amblyscirtes phylace* W.H. Edwards, 1878	29	USA (Texas)	Emmel and Trew 1973
175	*Amblyscirtes texanae* Bell, 1927	29	USA (Texas)	Emmel and Trew 1973
176	*Amblyscirtes vialis* (W. H. Edwards, 1862)	29	USA (Connecticut)	Maeki 1961
177	*Cymaenes* sp.	31	Tobago	Wesley and Emmel 1975
178	*Enosis immaculata immaculata* (Hewitson, 1868)	29	Ecuador	Kumagai et al. 2010
179	*Lerema accius* (Smith, 1797)	29 (2n=58)	USA (Texas)	Goodpasture 1976
		29	USA (Texas)	Emmel and Trew 1973
180	*Moeris vopiscus* (Herrich-Schäffer, 1869)	27	Peru	Kumagai et al. 2010
181	*Nastra lherminier* (Latreille, [1824])	30	USA (Connecticut)	Maeki 1961
182	*Thargella caura* (Plötz, 1882)	25	Brazil	de Lesse and Brown 1971
183	*Vettius coryna* (Hewitson, [1866])	31, ca32	Ecuador	de Lesse 1967a
184	*Vettius phyllus prona* Evans, 1955	26	Brazil	de Lesse and Brown 1971
185	*Vettius triangularis* (Hübner, [1831])	26	Brazil	Kumagai et al. 2010
**Tribe Hesperiini**				
186	*Asbolis capucinus* (Lucas, 1857)	48	USA (Florida)	Maeki 1961
187	*Cynea iquita* (Bell, 1941)	29	Argentina	de Lesse 1967a
188	*Hesperia comma* (Linnaeus, 1758)	28	Italy	de Lesse 1970c
		28	Lebanon	Larsen 1975
189	*Hesperia florinda* Butler, 1878	28 (2n=56)	Japan	Abe et al. 2006
190	*Hylephila fasciolata* (Blanchard, 1852)	29	Argentina	de Lesse 1967a
191	*Hesperia phyleus* (Drury, 1773)	29	Argentia	de Lesse 1967a
		29	USA (Florida)	Maeki 1961
192	*Hesperia signata* (Blanchard, 1852)	29	Chile	de Lesse 1967a
193	*Ochlodes ochraceus* (Bremer, 1861)	29 (2n=58)	Japan	Abe et al. 2006
		24	Japan	Maeki and Remington 1960
194	*Ochlodes sylvanoides* (Boisduval, 1852)	29	USA	Maeki 1961
195	*Ochlodes sylvanus* (Esper, 1777)	29	Finland	Federley 1938
		29	Croatia	Lorković 1941
196	*Ochlodes venatus* (Bremer et Grey, 1853) (as *sylvanus* Esper, 1777)	29 (2n=58)	Japan	Abe et al. 2006
197	*Oligoria maculata* (W. H. Edwards, 1865)	29	USA (Florida)	Maeki 1961
198	*Poanes hobomok hobomok* (Harris, 1862)	29	?USA	Lorković in Robinson 1971
199	*Poanes taxiles* (W. H. Edwards, 1881)	29	USA	Maeki 1961
200	*Poanes zabulon* (Boisduval et Le Conte, [1837]) (as *Polites zabulon*)	29	USA (Connecticut)	Maeki 1961
201	*Polites themistocles* (Latreille, [1824])	29	USA (Florida)	Maeki 1961
202	*Poanes vibex catilina* (Plötz, 1886)	29	Argentina	de Lesse 1967a
	*Poanes vibex praeceps* (Scudder, 1872)	27	USA (Texas)	Emmel and Trew 1973
	*Poanes vibex vibex* (Geyer, 1832)	29	USA (Florida)	Maeki 1961
203	*Wallengrenia egeremet* (Scudder, 1863)	28	USA (Texas)	Emmel and Trew 1973
204	*Wallengrenia otho curassavica* (Snellen, 1887)	28–30	USA (Texas)	Emmel and Trew 1973
205	*Wallengrenia premnas* (Wallengren, 1860)	27	Argentina	de Lesse 1967

### Detecting cryptic species using analysis of chromosomal differences

Recent years karyological data have been widely used in studies of butterfly taxonomy and in biodiversity research as main or additional chracters for detecting cryptic species (e.g. Dinca et al. 2011) and for synonymizing biological entities that were incorrectly described as distinct species (e.g. Vila et al. 2010). The family Hesperiidae is not excluded in this respect. In the genus *Gegenes* Hübner, [1819], two cryptic species *Gegenes pumilio* (n=24) and *Gegenes gambica* (n=41) were discovered through extensive chromosome analysis of different populations (de Lesse 1960, 1967b, Larsen 1982, Saitoh 1984).

In the genus *Pyrgus* Hübner, [1819], our unpublished chromosome data (see Table) were used to recognize and then to describe two morphologically similar species, *Pyrgus bolkariensis* and *Pyrgus aladaghensis* (De Prins and van der Poorten 1995).

Thus, interspecific chromosomal differences are useful for discovering and describing new cryptic species of Hesperiidae representing in such a way a powerful tool in biodiversity research.
